# Transforming Growth Factor α Evokes Aromatase Expression in Gastric Parietal Cells during Rat Postnatal Development

**DOI:** 10.3390/ijms25042119

**Published:** 2024-02-09

**Authors:** Hiroto Kobayashi, Akira Naito, Kyutaro Kawagishi

**Affiliations:** 1 Department of Anatomy and Structural Science, Faculty of Medicine, Yamagata University, 2-2-2 Iida-Nishi, Yamagata 990-9585, Japan; 2 Department of Rehabilitation, Faculty of Medical Science and Welfare, Tohoku Bunka Gakuen University, Sendai 981-8551, Japan

**Keywords:** stomach, extra-glandular steroidogenesis, differentiation, EGFR, ERK1+2, MAPK, estradiol, stem cell, weaning, aging

## Abstract

Estrogen, well known as a female hormone, is synthesized primarily by ovarian aromatase. However, extra-glandular tissues also express aromatase and produce estrogen. It is noteworthy that aromatase in gastric parietal cells begins expression around 20 days after birth and continues secreting considerable amounts of estrogen into the portal vein throughout life, supplying it to the liver. Estrogen, which is secreted from the stomach, is speculated to play a monitoring role in blood triglyceride, and its importance is expected to increase. Nevertheless, the regulatory mechanisms of the aromatase expression remain unclear. This study investigated the influence of transforming growth factor α (TGFα) on gastric aromatase expression during postnatal development. The administration of TGFα (50 μg/kg BW) to male Wistar rats in the weaning period resulted in enhanced aromatase expression and increased phosphorylated ERK1+2 in the gastric mucosa. By contrast, administration of AG1478 (5 mg/kg BW), a protein tyrosine kinase inhibitor with high selectivity for the epidermal growth factor receptor and acting as an antagonist of TGFα, led to the suppression of aromatase expression. In fact, TGFα expression in the gastric fundic gland isthmus began around 20 days after birth in normal rats as did that of aromatase, which indicates that TGFα might induce the expression of aromatase in the parietal cells concomitantly.

## 1. Introduction

Estrogen is a group of steroid hormones that includes estrone, estradiol, and estriol. Those hormones are synthesized by aromatase, and estradiol is the most bioactive form among estrogens. The granulosa cells of the ovary are well known as the tissue responsible for synthesizing estrogen. However, the expression of aromatase in the ovary fluctuates considerably during the estrus cycle, exhibiting notable upregulation during proestrus and minimal expression during other phases. Circulating estradiol levels in arterial blood also reflect this periodicity [1,2,3,4,5,6,7].

Moreover, aromatase is found in various extra-glandular tissues, contributing to estrogen production [8]. It is noteworthy that a considerable amount of estradiol production occurs in the gastric parietal cells of rats [9,10]. Although the stomach releases large amounts of estradiol into the portal vein, most of the estradiol either binds to hepatic estrogen receptors or undergoes enzymatic metabolism in the liver. This binding and metabolism are evident from the higher estradiol levels in portal blood than in arterial blood. It has long been estimated that estrogen secreted from the stomach contributes to the maintenance of hepatic function. However, a recent study highlighted the role of gastric aromatase in using circulating triglycerides as an energy source for estradiol production, suggesting the role of the stomach in monitoring triglyceride levels and shedding new light on the importance of estrogen in the gastric milieu [11]. Despite this accumulated knowledge, the regulatory mechanisms governing gastric aromatase expression remain elusive.

Gastrointestinal tract development and maturation are coordinated through a complex interaction of hormones, growth factors, milk-born molecules, luminal microbes, and genetic programs [12]. Especially, many parts of the gastrointestinal tract synthesize transforming growth factor alpha (TGFα) to enhance the epithelial cell migration and proliferation necessary to compensate for continual cell loss and to protect the integrity of the epithelium from lesions caused, for example, by low pH or oxidation [13]. TGFα is a growth factor that acts in an autocrine or paracrine. In addition to the growth function, TGFα binds to the epidermal growth factor receptor (EGFR) and contributes to differentiation, development, embryogenesis, tumorigenesis, and angiogenesis [13,14]. In the stomach, TGFα has also been known to play a role in increasing mucus and mucous cells, providing protection to the gastric mucosa, and inhibiting gastric acid secretion [15,16]. Recent studies have documented more detailed functions of TGFα in the stomach, which is produced and secreted by the progenitor cells of surface mucous cells and plays a key role in stimulating and regulating the differentiation into surface mucous cells within healthy adult mouse gastric tissues via the EGFR-ERK pathway [17].

The expression of the substances in the stomach is highly diversified. For example, during postnatal development of the rat stomach, H^+^/K^+^-ATPase β-subunit, the proton pump for secreting gastric acid, is identified and expressed in parietal cells on postnatal day 1. Additionally, pepsinogen in the chief cells and ghrelin in the A-like cells are identified by postnatal day 5. Aromatase emerges in the epithelial cells of the gastric fundic gland isthmus on approximately postnatal day 20 [18,19]. Then, aromatase increases gradually, subsequently reaching a plateau at around postnatal day 40, and remaining more or less stable thereafter [19]. However, the relationship between TGFα and aromatase in the developing stomach remains unclear. This study specifically examines TGFα, with the aim of elucidating the mechanism of aromatase expression in the stomach.

## 2. Results

### 2.1. Effects of TGFα Administration

#### 2.1.1. Morphological Changes in the Stomach

**Figure 1:**
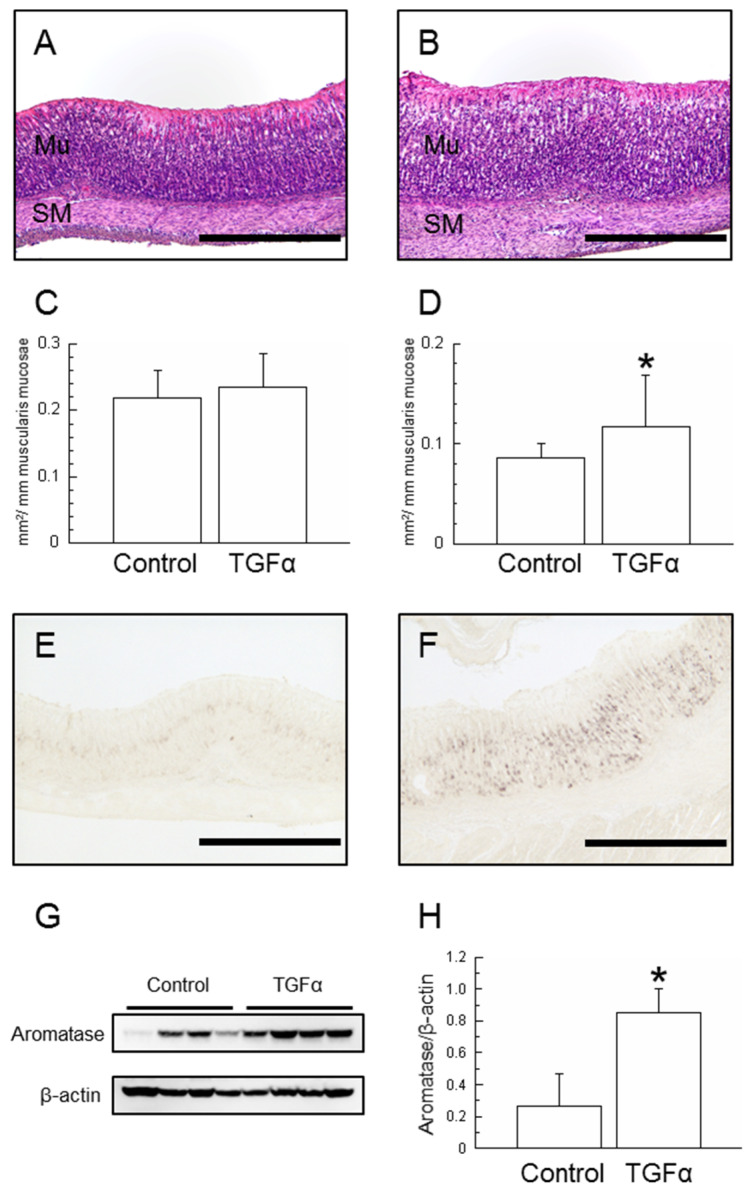
HE-stained sections of gastric mucosa (Control, (**A**); TGFα-administered, (**B**)) and histological image analysis of the area of the mucosal epithelium (**C**) and the smooth muscle layer (**D**) per 1 mm of the muscularis mucosae at day 21. Light photomicrographs of the gastric mucosa of the Control (**E**) and TGFα-administered rats (**F**) were immunostained with aromatase antibody. Western blot analysis of gastric aromatase protein was conducted. Aromatase (upper lane) and β-actin (lower lane) were detected by immunoblotting (**G**). Experiments were conducted by loading equal amounts of gastric mucosal proteins in each lane. Aromatase protein increased with TGFα administration (**H**). *n* = 4, mean ± S.D. Scale bars represent 500 μm. Mu, mucosal epithelium; SM, smooth muscle; *, *p* < 0.05 vs. Control.

After 6 days (until day 21) of administration of TGFα to weaning rats, significant increases in both body and gastric weight were found (*p* < 0.05, respectively, Table 1). Furthermore, a significant increase in gastric weight relative to body weight was found (*p* < 0.05). Histological hematoxylin- and eosin (HE)-stained sections of the gastric mucosa were used to measure the area of the mucosal epithelium and the smooth muscle layer per millimeter of the muscularis mucosae (Figure 1A,B). Although no significant differences were found in the mucosal layer, a significant increase was found in the smooth muscle layer in the TGFα-treated group (Figure 1C,D).

#### 2.1.2. Aromatase Expression in the Stomach

Immunohistochemistry using aromatase antibodies revealed that, in the control group, there were scattered aromatase positive cells in the proliferative zone. In the TGFα-treated group, a significant increase in aromatase positive cells was found (Figure 1E,F). The gastric mucosa homogenates displayed a single band with a molecular mass of 55 kDa by Western blotting, which is consistent with the molecular mass of aromatase (Figure 1G). The immunoblot bands revealed that aromatase protein levels were measured; a significant increase was found for the TGFα-treated group (*p* < 0.05, Figure 1H).

#### 2.1.3. ERK1+2 Expression in the Stomach

**Figure 2:**
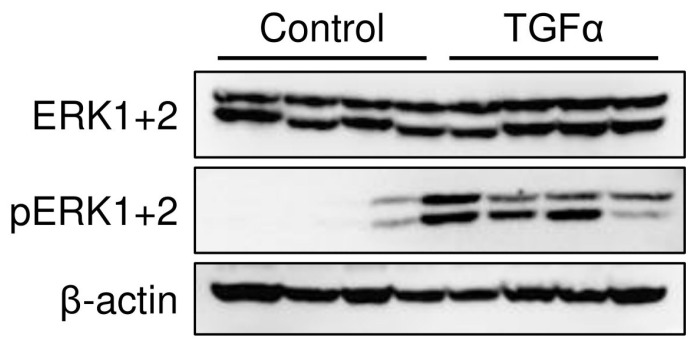
Western blot analysis of gastric ERK1+2 and phosphorylated ERK1+2 protein. TGFα administration increased the expression of pERK1+2 proteins in the stomach.

Because TGFα is known to bind to the EGFR and to activate the MAPK signaling pathway, we took measurements of ERK1+2 and phosphorylated ERK1+2 protein levels in the stomach, which are typically associated with proliferative signal transduction. The results revealed that TGFα administration enhanced the expression of phosphorylated ERK1+2 in the gastric mucosa (Figure 2).

### 2.2. TGFα Expression in the Stomach during Postnatal Development

**Figure 3:**
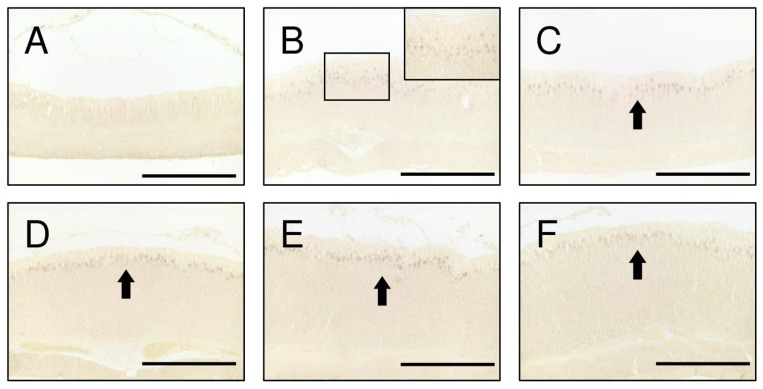
Stomach sections at day 15 (**A**), 20 ((**B**); box shows the marked area of (**B**)), 25 (**C**), 30 (**D**), 35 (**E**), and 40 (**F**) were immunostained with antibodies to TGFα. A few cells were stained slightly at 20 days (**B**). The number of immunostained cells and their immunostainabilities were greater after day 25 ((**C**–**F**), arrows). Scale bars represent 500 μm.

Investigations into the onset of TGFα expression in the gastric mucosa were conducted every 5 days from postnatal day 15 through day 40. The results revealed the absence of TGFα positive cells at day 15 (Figure 3A). However, at day 20, TGFα positive cells began to emerge in the proliferative zone of the gastric mucosa (Figure 3B). This expression persisted from postnatal day 25 through day 40 (Figure 3C–F). No TGFα positive cells were observed before day 15.

### 2.3. Effects of AG1478 Administration

**Figure 4:**
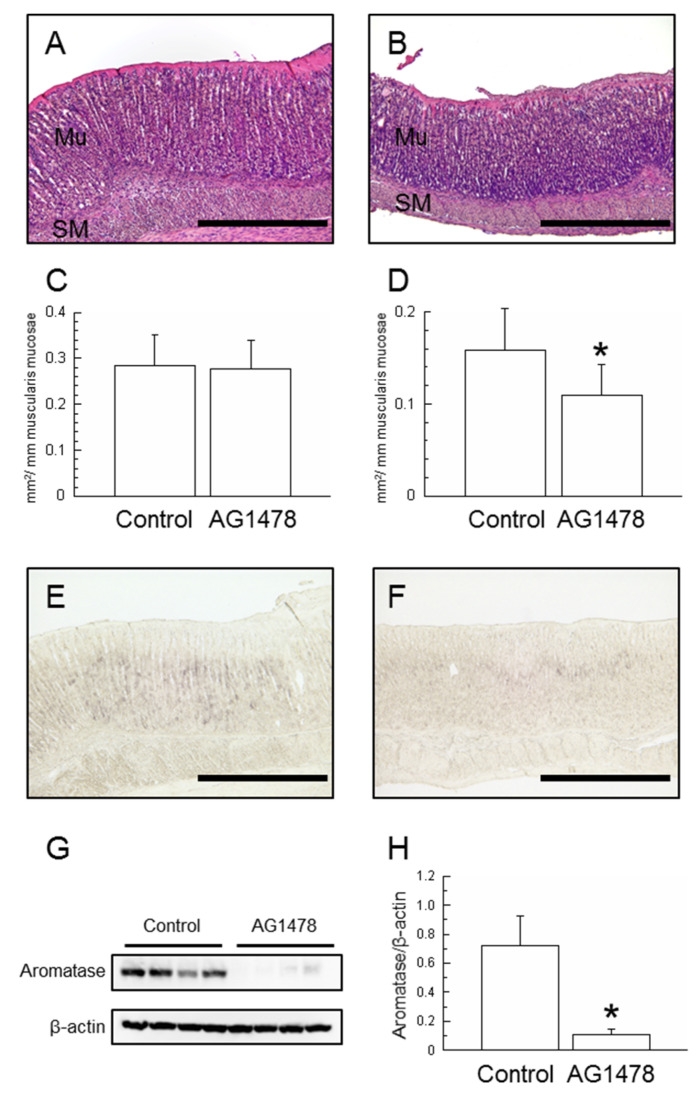
HE staining sections of gastric mucosa (Control, (**A**); AG1478-administered, (**B**)) and histological image analysis of the areas of the mucosal epithelium (**C**) and the smooth muscle layer (**D**) per 1 mm of the muscularis mucosae at day 25. Light photomicrographs of the gastric mucosa of the Control (**E**) and AG1478 administration (**F**) were immunostained with aromatase antibodies. Western blot analysis of gastric aromatase protein. Aromatase (upper lane) and β-actin (lower lane) were detected by immunoblotting (**G**). Aromatase protein decreased with AG1478 administration (**H**). *n* = 4, mean ± S.D. Scale bars represent 500 μm. Mu, mucosal epithelium; SM, smooth muscle; *, *p* < 0.05 versus Control.

After 9 days (day 25) of AG1478 administration to weaning rats, no significant difference was found in body weight (*p* = 0.1162, Table 2). However, stomach weight and gastric weight relative to body weight had each decreased significantly (*p* < 0.05, respectively, Table 2). The area of the mucosal epithelium and the smooth muscle layer were measured using the same method as that for TGFα administration (Figure 4A,B). Although no significant difference was found in the mucosal layer, the smooth muscle layer of the AG1478-treated group was found to have a significant decrease (Figure 4C,D).

Immunohistochemical staining with sections of the gastric mucosa revealed that aromatase positive cells in the control group were expressed broadly from the glandular neck region to the glandular body (Figure 4E). By contrast, their expression was inhibited in the AG1478-treated group, remaining confined to the proliferative zone in the gastric fundic isthmus (Figure 4F). The immunoblot bands revealed aromatase protein levels, as measured using Western blotting (Figure 4G). A significant decrease was found for the AG1478-treated group (*p* < 0.05, Figure 4H).

## 3. Discussion

The results of this study revealed that TGFα serves as the initiating substance for aromatase expression during postnatal development of the rat stomach. Although the importance of estrogen synthesized from the stomach has been suggested [11], this study is a crucially important investigation that elucidates the regulating factor of estrogen in the stomach.

Various tissues are known to produce TGFα, which exerts its effects in autocrine and paracrine manners [13]. Cells of the gastric mucosa, including parietal cells, which express aromatase, have EGFR. [17,20]. Gastric smooth muscle is also influenced by TGFα as a potent mitogenic factor [13,21]. In this study, TGFα administration was closely associated with increased body weight, stomach weight, and stomach weight per body weight (Table 1). These findings suggest that the augmentation of gastric weight was more pronounced than the increase in body weight. Furthermore, although the gastric mucosa area was unchanged, the smooth muscle area increased (Figure 1). By contrast, although no significant difference was found in body weight in the AG1478-treated group, both the gastric weight and the stomach weight per body weight were decreased (Table 2). AG1478 is a tyrosine kinase inhibitor specifically selective to EGFR [22]. The expression of TGFα in the gastric mucosa was maintained (Figure S1). Consequently, it is conceivable that TGFα administration stimulated the proliferation of smooth muscle and that AG1478 administration impeded the differentiation and growth of the stomach. These results of our study are consistent with those of earlier studies [13,21].

TGFα administration enhanced the expression of aromatase in the gastric mucosa (Figure 1) and phosphorylated ERK1+2 of MAPK signaling pathways (Figure 2). TGFα and EGFR, which upregulate MAPK signaling pathways in the gastric mucosa, accelerated those expressions by early weaning [19,23]. Natural rat weaning typically occurs gradually during postnatal day 14 through day 30, with an accelerated phase during day 18 through day 25 [24,25]. TGFα positive cells in the gastric mucosa began to emerge at approximately day 20 (Figure 3). This timing, in turn, suggests that TGFα expression is induced with weaning, triggering a cascade and leading to the induction of aromatase expression in parietal cells. Furthermore, AG1478 administration to weaning rats suppressed aromatase expression in the gastric mucosa (Figure 4). Weaning, regarded as one factor influencing TGFα expression, remains unclear, with many obscure aspects involving numerous complex factors [25]. Results of an earlier study suggest that early weaning upregulates TGFα expression [19]. Our preliminary findings indicate that mechanical stimulation to the stomach upon transitioning from milk to solid food is a contributing factor to aromatase expression. This supposition might be explained by the enhanced TGFα expression because of early weaning. Although TGFα has been elucidated as one factor contributing to the expression of aromatase in the postnatal developmental of the stomach, whether a similar mechanism exists in adult rats remains uncertain. In aged rats (24 months old), a decline in gastric aromatase has been observed [26]; TGFα expression is also attenuated compared with that at 3 months (Figure S2). These results are considered to be related to an increase in sensitivity to EGFR ligands in the gastric mucosa with aging [27]. From this finding, it can be inferred that some correlation might exist between gastric aromatase and TGFα, even in adults.

Furthermore, a significant increase in aromatase mRNA expression in the gastric mucosa was found following TGFα administration (Table S1). However, no significant difference was found in the estradiol levels in portal blood (Table S2), presumably because the normal amount of estradiol in the portal blood of adult rats is around 100 pg/mL [9,12]. In this study, aromatase in the gastric mucosa had just begun to be expressed and was close to the detection limit for estradiol measurement. The ambiguity might stem from the substrate of estradiol, or it could be due to an insufficient supply of aromatase. Further research is needed to address this aspect.

TGFα, a crucially important growth factor in various tissues, including the stomach, offers a promising avenue for gaining fresh insights into gastric development, protection, and function. If aromatase expression in the stomach as the extra-glandular tissue were found to be modulated through TGFα, then this finding could provide new perspectives on the interplay between reproductive hormones and the gastric system. The importance of unraveling this mechanism is underscored by its potential implications for our understanding of reproductive biology, endocrinology, and gastrointestinal physiology, offering promising directions for future research and clinical applications.

## 4. Materials and Methods

### 4.1. Animals

In accordance with earlier studies [28], TGFα (50 μg/kg BW, #201-18341; Fujifilm Wako Pure Chemical Corp., Osaka, Japan) diluted in 0.1% bovine serum albumin (BSA, # 013-15104; Fujifilm Wako Pure Chemical Corp.) in distilled water was administered i.p. once daily (8:00 a.m.) to Wistar male rats (Japan SLC, Inc., Shizuoka, Japan) of neonatal age from postnatal day 16 to day 21 (*n* = 6). Tissue collection was conducted on postnatal day 21 after the last TGFα injection. A control group was prepared, to which only the same amount of the BSA solution was administered (*n* = 6). To analyze the expression of TGFα in the stomach during postnatal development, we conducted experiments by collecting the stomachs of Wistar male rats on days 15, 20, 25, 30, 35, and 40. Furthermore, the protein tyrosine kinase inhibitor AG1478 (5 mg/kg BW, #S2728; Selleck Biotech, Yokohama, Japan), which exhibits high selectivity for the EGFR [22], was used to inhibit TGFα during the postnatal development process according to an earlier study [14]. After dissolving AG1478 in dimethyl sulfoxide (DMSO, final concentration was 0.1%, #049-07213; Fujifilm Wako Pure Chemical Corp.), it was suspended in olive oil (#150-00276; Fujifilm Wako Pure Chemical Corp.) and was administered i.p. once daily (8:00 a.m.) on postnatal days 16–24. Tissue collection occurred on postnatal day 25. The control group rats received an equivalent volume of olive oil with dissolved DMSO only, administered once daily.

Rats were anesthetized using a combination of anesthetic agents of three types: medetomidine hydrochloride (0.15 mg/kg body weight; Nippon Zenyaku Kogyo Co., Ltd., Fukushima, Japan), midazolam (2 mg/kg body weight; Astellas Pharma Inc., Tokyo, Japan), and butorphanol tartrate (2.5 mg/kg body weight; Meiji Seika Pharma Co., Ltd., Tokyo, Japan). All animals were euthanized by exsanguination. The stomach was excised promptly at each age, rinsed with phosphate-buffered saline (PBS), weighed, and stored at −80 °C until further analysis. The animals were housed in a room with a 12 h light and 12 h dark cycle (lights on from 6:00 a.m. to 6:00 p.m.) under controlled temperature conditions. They were provided a standard pellet diet (Oriental Yeast Co., Ltd., Tokyo, Japan) and had access to tap water ad libitum, including nursing mother rats. All procedures were conducted at 9:00 a.m., following institutional guidelines and with approval from the animal research ethical committee of Yamagata University.

### 4.2. Histological Analysis

For HE and immunohistochemical staining, the stomachs were fixed overnight at 4 °C in Bouin’s solution without acetic acid. Subsequently, the tissues underwent dehydration through a graded ethanol series and were embedded in embedding media (Paraplast; Sigma-Aldrich Japan K.K., Tokyo, Japan). A histological examination of the stomach was conducted conventionally with HE staining to verify its histological structure. In parallel, adjacent sections were subjected to immunostaining using the peroxidase-labeled antibody method, employing antibodies against aromatase (1:1000, #MCA2077S; AbD Serotec, Oxford, UK). Immunostaining for the postnatal development of the stomach was performed using the antibody against TGFα (1:700, #NBP2-34683; Novus Biologicals Inc., Littleton, CO, USA). Following deparaffinization and hydrophilization, sections were incubated overnight at 37 °C with the primary antibody. Then, they were reacted for an hour at 37 °C with the anti-mouse IgG conjugated with horseradish peroxidase (1:10, #424151, Histofine, Simple Stain MAX-PO [MULTI]; Nichirei Corp., Tokyo, Japan) after rinsing with PBS. Development was achieved using a chromogen solution consisting of 0.002% 3,3′-diaminobenzidine tetrachloride and 1% H_2_O_2_ in 20 mM Tris buffer (pH 7.4), supplemented with 1 mM ammonium nickel (II) sulfate hexahydrate. As a negative control, nearly adjacent sections were incubated without the primary antibody. Then, they were incubated with the secondary antibody. No labeling was confirmed in these control sections. Images were captured using a camera (DFC7000T; Leica Microsystems GmbH, Wetzlar, Germany) attached to a microscope (DM2500LED; Leica Microsystems GmbH). More than ten micrographs were taken from each rat stomach section. These images were subsequently analyzed using software (Image J 1.48v; National Institutes of Health, Bethesda, MD, USA). Consequently, specific areas of mucosal epithelium or muscularis mucosae were analyzed. The areas of mucosal epithelium or muscularis mucosae were calculated and expressed in appropriate units of muscularis mucosae: mm^2^/1 mm.

### 4.3. Western Blotting

The stomach tissue (*n* = 4) was homogenized using RIPA buffer (Fujifilm Wako Pure Chemical Corp.) supplemented with a protease inhibitor (cOmplete™ Protease Inhibitor Cocktail, Roche Diagnostics Corp., Basel, Switzerland). The protein concentration was determined using a TaKaRa BCA Protein Assay kit (Takara Bio Inc., Kusatsu, Japan). For immunoblotting, 20 μg of total protein was separated on a 10% SDS-PAGE gel. The separated protein samples were then transferred onto PVDF membranes with subsequent blocking with 4% non-fat Skim Milk Powder (Fujifilm Wako Pure Chemical Corp.) in Tris Buffered Saline with 0.1% Tween 20 (#103168; MP Biomedicals, Solon, OH, USA). Membranes were incubated with primary antibodies against aromatase (1:10,000; AbD Serotec, Kidlington, UK), ERK1 + ERK2 (1:1000; #ab184699; Abcam plc., Cambridge, UK), ERK1 (phospho T202) + ERK2 (phosphor T185) (1:1000; #ab201015; Abcam plc.), and β-actin (1:100,000, #sc-47778; Santa Cruz Biotechnology Inc., Dallas, TX, USA). Following incubation with anti-mouse or rabbit IgG, HRP-linked antibodies (1:3000, #7074 or #7076; Cell Signaling Technology Inc., Danvers, MA, USA), the blots were visualized using ImmunoStar LD (Fujifilm Wako Pure Chemical Corp.). As a negative control, membranes were incubated without the primary antibody under the same condition. No signaling was confirmed.

### 4.4. Statistical Analysis

Data were subjected to statistical analysis using Student’s *t*-test, conducted using software (StatView Version 5.0; Hulinks Inc., Tokyo, Japan). Significance was inferred for any *p*-value less than 0.05.

## 5. Conclusions

The expression of aromatase in the postnatal development of the rat stomach appears to be influenced by TGFα, which is expressed around postnatal day 20 and appears to be regulated through the MAPK signaling pathway.

## Figures and Tables

**Table 1:** Effects of TGFα administration on body and stomach weight at day 21.

	Control	TGFα
Body weight (g)	34.0 ± 1.7	42.4 ± 1.3 *
Stomach weight (mg)	251.1 ± 14.4	350.7 ± 36.2 *
Stomach weight ratio (mg/100 g BW)	745.8 ± 29.0	836.9 ± 82.4 *

*n* = 6, mean ± S.D.; *, *p* < 0.05.

**Table 2:** Effects of AG1478 administration on body and stomach weight at day 25.

	Control	AG1478
Body weight (g)	55.8 ± 5.1	51.7 ± 1.0
Stomach weight (mg)	342.3 ± 31.1	307.7 ± 6.0 *
Stomach weight ratio (mg/100 g BW)	613.6 ± 14.2	594.9 ± 5.8 *

*n* = 6, mean ± S.D.; *, *p* < 0.05.

## Data Availability

Data is contained within the article and Supplementary Materials.

## Supplementary Materials

The following supporting information can be downloaded at https://www.mdpi.com/article/10.3390/ijms25042119/s1.
