# Supplementary material for: Transforming Growth Factor α Evokes Aromatase Expression in Gastric Parietal Cells during Rat Postnatal Development
Source: Int J Mol Sci. 2024 Feb 9;25(4):2119. doi: 10.3390/ijms25042119 (PMC10889205; doi:10.3390/ijms25042119)
Supplement: Supplementary file 1 [file ijms-25-02119-s001.zip › ijms-2818234-supplementary.pdf]

Supplemental data

Figure S1

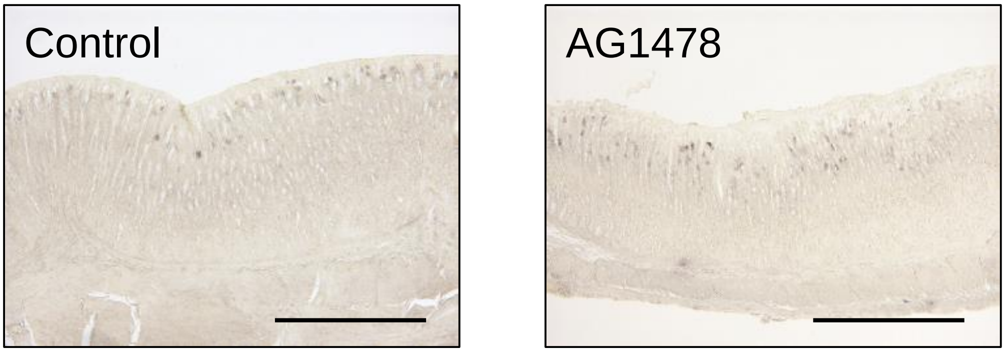

Light photomicrographs of the gastric mucosa of Control and AG1478 administration group at day 25 were immunostained with antibodies to TGFα under the same condition as outlined in the Materials and Methods section. TGFα expression is maintained in AG1478 administration group. Scale bars are provided to indicate 500 μm.

Figure S2

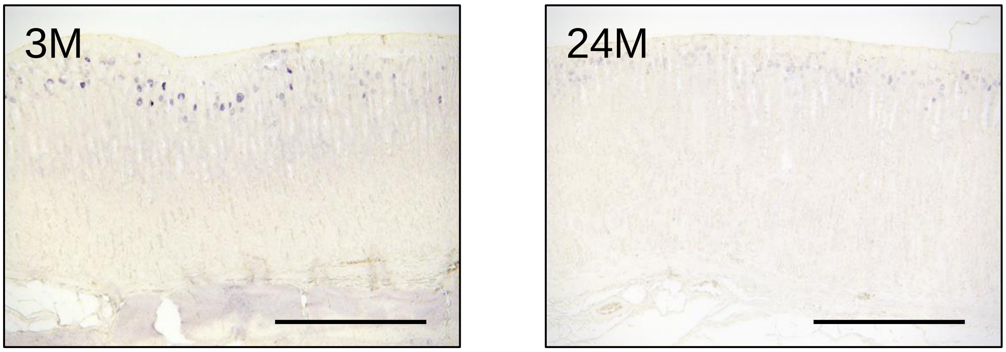

Light photomicrographs of the gastric mucosa of 3 months and 24 months old rat were immunostained with antibodies to TGFα under the same condition as outlined in the Materials and Methods section. Although TGFα expression was maintained at 3 months old, it was attenuated at 24 months old. Scale bars are provided to indicate 500 μm.

Supplemental data

Table S1.

The relative *Cyp19a1* mRNA content in the gastric mucosa at day 21

	Control	TGFα
Cyp19a1 mRNA	1.05 ± 0.36	3.25 ± 0.56 *

The *Cyp19a1* mRNA expression of TGFα was higher than Control group. Primer sequences used were the following: aromatase (*Cyp19a1*, GenBank accession no. NM_017085) forward, 5′-ATTGGCATGCACGAGAATGG-3′; reverse, 5′-TGCTGCTTGATGGATTCCAC-3′. n = 4, Mean ± S.D., *: $p < 0.05$.

Table S2.

Estradiol concentrations in the portal vein at day 21

	Control	TGFα
Estradiol (pg/mL)	6.95 ± 2.77	4.03 ± 4.18

There was no significant difference in the estradiol levels. n = 6, Mean ± S.D.
